# Reminiscence therapy care program as a potential nursing intervention to relieve anxiety, depression, and quality of life in older papillary thyroid carcinoma patients: A randomized, controlled study

**DOI:** 10.3389/fpsyg.2022.1064439

**Published:** 2022-11-24

**Authors:** Li Chen, Xianguang Yang, Xiukun Ren, Yufeng Lin

**Affiliations:** ^1^Department of Head and Neck Surgery, Harbin Medical University Cancer Hospital, Harbin, China; ^2^Clean Operating Department, Harbin Medical University Cancer Hospital, Harbin, China

**Keywords:** reminiscence therapy care program, anxiety and depression, quality of life, survival profile, older papillary thyroid carcinoma patients

## Abstract

**Objective:**

Reminiscence therapy (RT) ameliorates psychological problems and quality of life in cancer patients. However, no study reports its effect on older papillary thyroid carcinoma (PTC) patients. This study intended to investigate the effect of the RT-care program (CP) on anxiety, depression, quality of life, and survival in older PTC patients.

**Methods:**

Eighty-six postoperative older PTC patients were enrolled and randomly assigned to RT-CP group (N = 44) and usual (U)-CP group (N = 42) as a 1:1 ratio for a 6-month intervention. Hospital Anxiety and Depression Scale (HADS) and Quality of Life Questionnaire-Core 30 (QLQ-C30) scores were assessed at baseline, month (M)1, M2, M4, and M6.

**Results:**

HADS and QLQ-C30 scores at baseline were not different between two groups. Additionally, HADS-anxiety score at M6 (*p* = 0.029), and HADS-depression score at M2 (*p* = 0.030), M4 (*p* = 0.029), M6 (*p* = 0.012) were reduced in RT-CP group versus U-CP group. Meanwhile, anxiety and depression rates from M1 to M6 were slightly decreased in RT-CP group versus U-CP group but did not reach statistical significance. Furthermore, depression severity at M6 was reduced in RT-CP group versus U-CP group (*p* = 0.049). Besides, QLQ-C30 global health status was increased at M2 (*p* = 0.023) and M6 (*p* = 0.033), QLQ-C30 function score was elevated at M2 (*p* = 0.040) and M4 (*p* = 0.035), while QLQ-C30 symptom score was decreased at M2 (*p* = 0.046) in RT-CP group versus U-CP group. Moreover, disease-free survival and overall survival were not different between two groups.

**Conclusion:**

RT-CP may be a potential intervention for ameliorating anxiety, depression, and quality of life in older PTC patients.

## Introduction

Papillary thyroid carcinoma (PTC) is the predominant form of thyroid cancer, which accounts for nearly 85% of all thyroid cancer cases (Megwalu and Moon, 2022). Currently, the primary treatment strategy for PTC patients is surgery (including lobectomy, thyroidectomy, neck dissection, etc.; McDow et al., 2021; Ngo et al., 2021; Sanabria et al., 2022); followed by thyroid hormone therapy, radioactive iodine therapy, targeted therapy, etc. (Kim et al., 2020; Hescheler et al., 2021; Chan and Kwong, 2022). Benefiting from the advancements of these treatments, the prognosis of PTC patients is favorable with a 10-year overall survival (OS) rate more than 90% (Ito et al., 2018; Harries et al., 2020; Jukic et al., 2022). However, PTC patients usually suffer from a huge psychological burden and the PTC-induced thyroid hormones dysregulation may aggravate the psychological issues, including anxiety, depression, etc., as well as reduce the quality of life in PTC patients (Banihashem et al., 2020; Baksi and Pradhan, 2021; Liu and Brent, 2021). Meanwhile, older PTC patients usually possess a worse prognosis compared to younger patients, which may also affect their mental health and quality of life (Kaliszewski et al., 2020; Sun et al., 2020). Therefore, it is imperative to explore effective strategies to improve psychological health and quality of life in older PTC patients.

Reminiscence therapy (RT) is a kind of psychotherapy aiming to ameliorate the psychological status and quality of life by recalling and sharing wonderful past memories, which is generally applied to patients with neurological diseases, such as Alzheimer’s disease (AD), dementia, acute ischemic stroke (AIS), etc. (Hsiao et al., 2020; Li et al., 2020; Cheng et al., 2021; Li and Liu, 2022; Perez-Saez et al., 2022). At present, this intervention is also widely used in cancer patients to improve their mental health and quality of life (Liu and Li, 2021; Zhang et al., 2021). For instance, a study reports that RT is effective in decreasing anxiety and improving the quality of life in gastric cancer patients (Zhang et al., 2021). Meanwhile, RT is also helpful in ameliorating anxiety, depression, and quality of life in non-small cell lung cancer (NSCLC) patients (Liu and Li, 2021). Moreover, anxiety, depression, and quality of life are all relieved, and OS is also prolonged to some extent in colorectal cancer patients receiving RT compared with those receiving control care (Zhou and Sun, 2021). Therefore, it could be speculated that RT may be also effective in improving mental health and quality of life in older PTC patients. Nevertheless, the effect of RT in these patients has not been investigated yet.

Accordingly, this randomized, controlled study designed an RT-care program (RT-CP), intending to investigate the effect of RT-CP on psychological health and quality of life in older PTC patients.

## Materials and methods

### Patients

A total of 86 older PTC patients who received tumor resection between February 2019 and October 2021 were continuously included in this randomized, controlled study. The inclusion criteria contained: (a) pathologically confirmed as PTC; (b) age ≥ 60 years; (c) received tumor resection; (d) capable of the assessment required in the study; and (e) had willingness for participation and regular follow-up. The exclusion criteria contained: (a) complicated with other primary carcinomas or malignancies; (b) concomitant with neurodegenerative disease, or severe cognitive impairment; (c) patients who had documented diagnoses of anxiety and depression before surgery; and (d) incapable of normal communication. The study was permitted by the Ethics Committee of Harbin Medical University Cancer Hospital. Each patient signed the informed consent.

### Random assignment

Following the enrollment, eligible patients were assigned at random (1:1 ratio). The block randomization method was used to generate a random assignment table with a block size of 4. Random grouping information for each eligible patient was sealed in an opaque envelope that corresponded to the patient’s enrollment number. Based on the enrollment number, eligible patients were given the opaque envelope and then assigned to the appropriate group. As a result, 44 patients were assigned to the reminiscence therapy care program (RT-CP) and 42 patients were assigned to the usual care program (U-CP).

### Intervention

For patients in the U-CP group, U-CP was carried out after discharge at the rehabilitation center of our hospital once every 2 weeks for 6 months. Each U-CP lasted 120 min, which mainly included health education, standardized follow-up, and answering questions. The health education included an introduction to PTC, management of postoperative complications, rehabilitation guidance, self-monitoring, precautions, medication management, diet and lifestyle management, daily exercise, and mental health.

For patients in the RT-CP group, RT-CP was performed at the rehabilitation center of our hospital in small groups (7–10 patients who were recruited in the same or adjacent month for per group) once every 2 weeks for 6 months, starting from the first month after discharge. Each RT-CP lasted 120 min and consisted of two elements, which were health education and reminiscence therapy. The health education contained an introduction to PTC, management of postoperative complications, rehabilitation guidance, self-monitoring, precautions, medication management, diet and lifestyle management, daily exercise, and mental health. The reminiscence therapy contained the following 12 topics: (a) a brief self-introduction; (b) sharing an interesting childhood story; (c) sharing a memorable school story; (d) sharing scenery of hometown; (e) sharing favorite food of hometown; (f) sharing memorable travel experience; (g) sharing professional experiences; (h) sharing a personal hobby; (i) sharing a favorite sport; (j) sharing a favorite celebrity or star; (k) sharing a favorite book or music; and (l) review and summarization.

### Evaluation and follow-up

At discharge (M0), 1st month (M1), 2nd month (M2), 4th month (M4), and 6th (M6) after discharge, the Hospital Anxiety and Depression Scale (HADS) and Quality of Life Questionnaire-Core 30 (QLQ-C30) scores were assessed. The HADS score was adopted to evaluate anxiety and depression, with no being 0–7 scores, mild being 8–10 scores, moderate being 11–14 scores, and severe being 15–21 scores (Zigmond and Snaith, 1983). The QLQ-C30 score was adopted to evaluate the quality of life (Aaronson et al., 1993). Additionally, after the 6-month intervention, the patients received standardized follow-up until June 2022. Then, the survival outcomes including disease-free survival (DFS) and OS were imputed.

### Statistics

The sample size was estimated based on the hypothesis that the mean value HADS for anxiety (HADS-A) score at M6 was 4.0 with a standard deviation (SD) < 3.0 in the RT-CP group and 6.0 with an SD < 3.0 in the U-CP group, respectively (Nakamura et al., 2020). The significance level was set as 5%, and the power was set as 80%, as a result, the minimum sample size was 36 in each group. Considering that 15% of patients might be lost to follow-up, the sample size was adjusted to 42 in each group. SPSS statistical software (v.26.0, IBM, America) was used for data analysis. GraphPad Prism software (v.7.0, GraphPad Software Inc., America) was used for graph making. Comparisons between the groups were determined by the *Χ*^2^ test, Student *t*-test, or Wilcoxon rank sum test. DFS and OS were shown using Kaplan–Meier curves and analyzed by log-rank test. *p* < 0.05 was considered significant.

## Results

### Study flow

**Figure 1:**
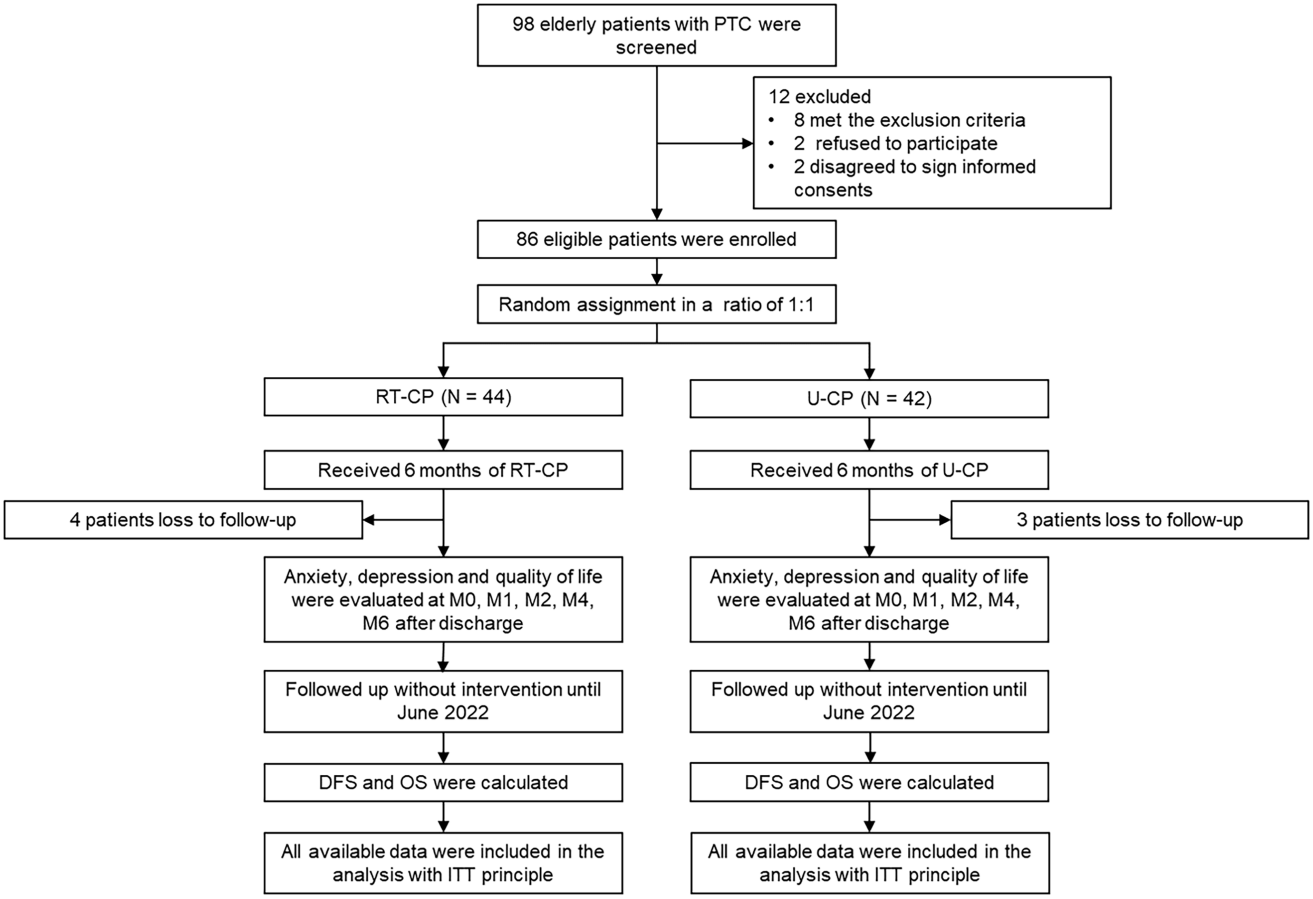
Study flow chart.

Totally, 98 older PTC patients were screened in this study, then 12 patients were excluded, containing 8 patients who met the exclusion criteria, 2 patients who refused to participate, and 2 patients who disagreed to sign informed consent. Subsequently, 86 eligible patients were enrolled and randomly assigned to the RT-CP group (*N* = 44; receiving RT-CP for 6 months) and the U-CP group (*N* = 42; receiving U-CP for 6 months) in a 1:1 ratio. During the follow-up period, 4 patients lost to follow-up in the RT-CP group; while 3 patients lost to follow-up in the U-CP group. In both the RT-CP group and the U-CP group, anxiety, depression, and quality of life were evaluated at M0, M1, M2, M4, and M6 after discharge. Then patients were followed up without intervention until June 2022, during which DFS and OS were calculated. Finally, all available data were included in the analysis with the intention-to-treat (ITT) principle (Figure 1).

### Comparison of baseline characteristics between RT-CP group and U-CP group

The mean age of older PTC patients in the RT-CP group and U-CP group was 64.3 ± 3.3 years and 65.5 ± 3.4 years (*p* = 0.102), respectively. Meanwhile, there were 14 (31.8%) males and 30 (68.2%) females in the RT-CP group, as well as 15 (35.7%) males and 27 (64.3%) females in the U-CP group (*p* = 0.702). Other clinical properties (including tumor size, extrathyroidal invasion, pathological-tumor-node-metastasis (pTNM) stage, and postoperative radioiodine) and demographical features (including smoking, educational level, marital status, employment status, and location) were not different between the RT-CP group and U-CP group either (all *p* > 0.05). Furthermore, the HADS-A score (*p* = 0.704), anxiety rate (*p* = 0.950), HADS-D score (*p* = 0.480), depression rate (*p* = 0.542), QLQ-C30 global health status score (*p* = 0.891), QLQ-C30 functions score (*p* = 0.830), and QLQ-C30 symptoms score (*p* = 0.843) were also not different between two groups at baseline. Notably, the administration of soporifics (*p* = 0.541), anxiolytics (*p* = 0.521), and antidepressants (*p* = 0.511) was not different between the two groups. Specifically, 12 (27.3%) patients in the RT-CP group used soporifics and 14 (33.3%) patients in the U-CP group applied this drug. Meanwhile, 8 (18.2%) patients in the RT-CP group administrated anxiolytics, and 10 (23.8%) patients in the U-CP group applied that. In addition, 7 (15.9%) patients in the RT-CP group used antidepressants, and 9 (21.4%) patients in the U-CP group applied this drug (Table 1).

**Table 1:** Comparison of baseline characteristics among the older PTC patients.

Characteristics	RT-CP (*N* = 44)	U-CP (*N* = 42)	*p-*value
Age (years)^a^	64.3 ± 3.3	65.5 ± 3.4	0.102
Gender^b^			0.702
Male	14 (31.8)	15 (35.7)	
Female	30 (68.2)	27 (64.3)	
Smoker^b^			0.535
Yes	10 (22.7)	12 (28.6)	
No	34 (77.3)	30 (71.4)	
Education level^b^			0.294
Primary school or less	7 (15.9)	7 (16.7)	
Middle or high school	21 (47.7)	26 (61.9)	
Undergraduate or above	16 (36.4)	9 (21.4)	
Marital status^b^			0.918
Married	36 (81.8)	34 (81.0)	
Single/divorced/widowed	8 (18.2)	8 (19.0)	
Employment status^b^			1.000
Unemployed	44 (100.0)	42 (100.0)	
Employed	0 (0.0)	0 (0.0)	
Location^b^			0.708
Rural	9 (20.5)	10 (23.8)	
Urban	35 (79.5)	32 (76.2)	
Tumor size^b^			0.650
≤4 cm	23 (52.3)	24 (57.1)	
>4 cm	21 (47.7)	18 (42.9)	
Extrathyroidal invasion^b^			0.201
No	17 (38.6)	22 (52.4)	
Yes	27 (61.4)	20 (47.6)	
pTNM stage^b^			0.278
I	1 (2.3)	2 (4.8)	
II	22 (50.0)	25 (59.5)	
III	15 (34.1)	10 (23.8)	
IV	6 (13.6)	5 (11.9)	
Postoperative radioiodine^b^			0.202
No	15 (34.1)	20 (47.6)	
Yes	29 (65.9)	22 (52.4)	
Soporifics^b^			0.541
No	32 (72.7)	28 (66.7)	
Yes	12 (27.3)	14 (33.3)	
Anxiolytics^b^			0.521
No	36 (81.8)	32 (76.2)	
Yes	8 (18.2)	10 (23.8)	
Antidepressants^b^			0.511
No	37 (84.1)	33 (78.6)	
Yes	7 (15.9)	9 (21.4)	
HADS-A score^a^	7.0 ± 2.9	7.2 ± 3.4	0.704
Anxiety^b^			0.950
No	28 (63.6)	27 (64.3)	
Yes	16 (36.4)	15 (35.7)	
HADS-D score^a^	6.6 ± 2.8	7.0 ± 3.2	0.480
Depression^b^			0.542
No	31 (70.5)	27 (64.3)	
Yes	13 (29.5)	15 (35.7)	
QLQ-C30 global health status score^a^	62.0 ± 12.8	61.5 ± 17.6	0.891
QLQ-C30 functions score^a^	63.7 ± 14.9	63.0 ± 17.4	0.830
QLQ-C30 symptoms score^a^	39.7 ± 14.9	40.4 ± 18.8	0.843

The *p*-values were based on *t*-tests for normal continuous variables, Chi-square test for unordered categorical variables, and nonparametric rank sum test for ordered categorical variables or non-normal categorical variables. PTC, papillary thyroid carcinoma; RT-CP, reminiscence therapy care program; U-CP, usual care program; pTNM, pathological-tumor-node-metastasis; HADS-A, Hospital Anxiety and Depression Scale for anxiety; HADS-D, Hospital Anxiety and Depression Scale for depression; QLQ-C30, Quality of Life Questionnaire-Core 30. ^a^Variables described by mean ± SD; ^b^Variables described by No. (%).

### Comparison of anxiety and depression between RT-CP group and U-CP group

**Figure 2:**
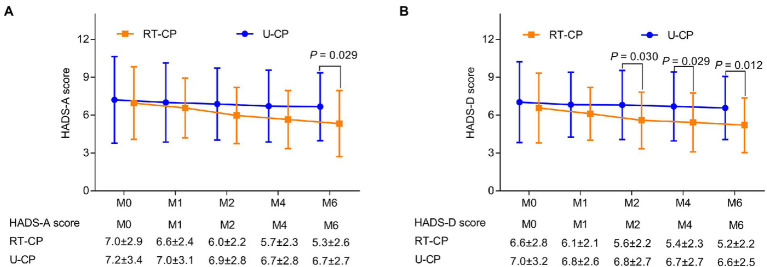
Comparison of Hospital Anxiety and Depression Scale (HADS) score at different time points between RT-CP group and U-CP group. HADS-A score at M6 was reduced in the RT-CP group in contrast with the U-CP group **(A)**; HADS-D score at M2, M4, and M6 was decreased in the RT-CP group in comparison with the U-CP group **(B)**.

HADS-A score was reduced at M6 in the RT-CP group in contrast to the U-CP group (5.3 ± 2.6 vs. 6.7 ± 2.7; *p* = 0.029), while it was not different at M0 (7.0 ± 2.9 vs. 7.2 ± 3.4), M1 (6.6 ± 2.4 vs. 7.0 ± 3.1), M2 (6.0 ± 2.2 vs. 6.9 ± 2.8), and M4 (5.7 ± 2.3 vs. 6.7 ± 2.8) between the RT-CP group and the U-CP group (all *p* > 0.05; Figure 2A). HADS-D score was decreased at M2 (5.6 ± 2.2 vs. 6.8 ± 2.7; *p* = 0.030), M4 (5.4 ± 2.3 vs. 6.7 ± 2.7; *p* = 0.029), and M6 (5.2 ± 2.2 vs. 6.6 ± 2.5; *p* = 0.012) in the RT-CP group by comparison with the U-CP group, whereas it was not different at M0 (6.6 ± 2.8 vs. 7.0 ± 3.2) and M1 (6.1 ± 2.1 vs. 6.8 ± 2.6) between the RT-CP group and U-CP group (both *p* > 0.05; Figure 2B).

Anxiety rate was numerically decreased at M1 (34.1% vs. 42.9%), M2 (29.3% vs. 39.0%), M4 (25.0% vs. 38.5%), and M6 (20.5% vs. 35.9%) in the RT-CP group by contrast to the U-CP group, but did not reach statistical significance (all *p* > 0.05). Depression rate was slightly reduced at M1 (22.7% vs. 33.3%), M2 (17.1% vs. 31.7%), M4 (17.5% vs. 30.8%), and M6 (12.8% vs. 30.8%) in the RT-CP group compared to the U-CP group, but did not reach statistical significance (all *p* > 0.05; Table 2).

**Table 2:** Comparison of anxiety and depression rate among the older PTC patients.

Items	Anxiety rate^a^			Depression rate^a^		
	RT-CP	U-CP	*p*-value	RT-CP	U-CP	*p*-value
M0	16 (36.4)	15 (35.7)	0.950	13 (29.5)	15 (35.7)	0.542
M1	15 (34.1)	18 (42.9)	0.403	10 (22.7)	14 (33.3)	0.273
M2	12 (29.3)	16 (39.0)	0.352	7 (17.1)	13 (31.7)	0.123
M4	10 (25.0)	15 (38.5)	0.198	7 (17.5)	12 (30.8)	0.168
M6	8 (20.5)	14 (35.9)	0.131	5 (12.8)	12 (30.8)	0.055

PTC, papillary thyroid carcinoma; RT-CP, reminiscence therapy care program; U-CP, usual care program; M0, discharge after operation; M1, 1 month discharge after operation; M2, 2 months discharge after operation; M4, 4 months discharge after operation; M6, 6 months discharge after operation. ^a^Variables described by No. (%).

Anxiety severity was not different at M0, M1, M2, M4, and M6 between the RT-CP group and the U-CP group (all *p* > 0.05). Depression severity was reduced at M6 in the RT-CP group compared with the U-CP group (*p* = 0.049). However, it was not different at M0, M1, M2, and M4 between the two groups (all *p* > 0.05; Table 3).

**Table 3:** Comparison of anxiety and depression severity among the older PTC patients.

Items	RT-CP				U-CP				*p*-value
	No^a^	Mild^a^	Moderate^a^	Severe^a^	No^a^	Mild^a^	Moderate^a^	Severe^a^	
Anxiety									
M0	28 (63.6)	11 (25.0)	4 (9.1)	1 (2.3)	27 (64.3)	7 (16.7)	7 (16.7)	1 (2.4)	0.847
M1	29 (65.9)	13 (29.5)	2 (4.5)	0 (0.0)	24 (57.1)	11 (26.2)	7 (16.7)	0 (0.0)	0.247
M2	29 (70.7)	11 (26.8)	1 (2.4)	0 (0.0)	25 (61.0)	12 (29.3)	4 (9.8)	0 (0.0)	0.275
M4	30 (75.0)	9 (22.5)	1 (2.5)	0 (0.0)	24 (61.5)	11 (28.2)	4 (10.3)	0 (0.0)	0.157
M6	31 (79.5)	6 (15.4)	2 (5.1)	0 (0.0)	25 (64.1)	10 (25.6)	4 (10.3)	0 (0.0)	0.131
Depression									
M0	31 (70.5)	9 (20.5)	4 (9.1)	0 (0.0)	27 (64.3)	10 (23.8)	4 (9.5)	1 (2.4)	0.520
M1	34 (77.3)	8 (18.2)	2 (4.5)	0 (0.0)	28 (66.7)	10 (23.8)	4 (9.5)	0 (0.0)	0.252
M2	34 (82.9)	6 (14.6)	1 (2.4)	0 (0.0)	28 (68.3)	8 (19.5)	5 (12.2)	0 (0.0)	0.098
M4	33 (82.5)	6 (15.0)	1 (2.5)	0 (0.0)	27 (69.2)	8 (20.5)	4 (10.3)	0 (0.0)	0.143
M6	34 (87.2)	5 (12.8)	0 (0.0)	0 (0.0)	27 (69.2)	10 (25.6)	2 (5.1)	0 (0.0)	0.049

PTC, papillary thyroid carcinoma; RT-CP, reminiscence therapy care program; U-CP, usual care program; M0, discharge after operation; M1, 1 month discharge after operation; M2, 2 months discharge after operation; M4, 4 months discharge after operation; M6, 6 months discharge after operation. ^a^Variables described by No. (%)

### Comparison of quality of life between RT-CP group and U-CP group

**Figure 3:**
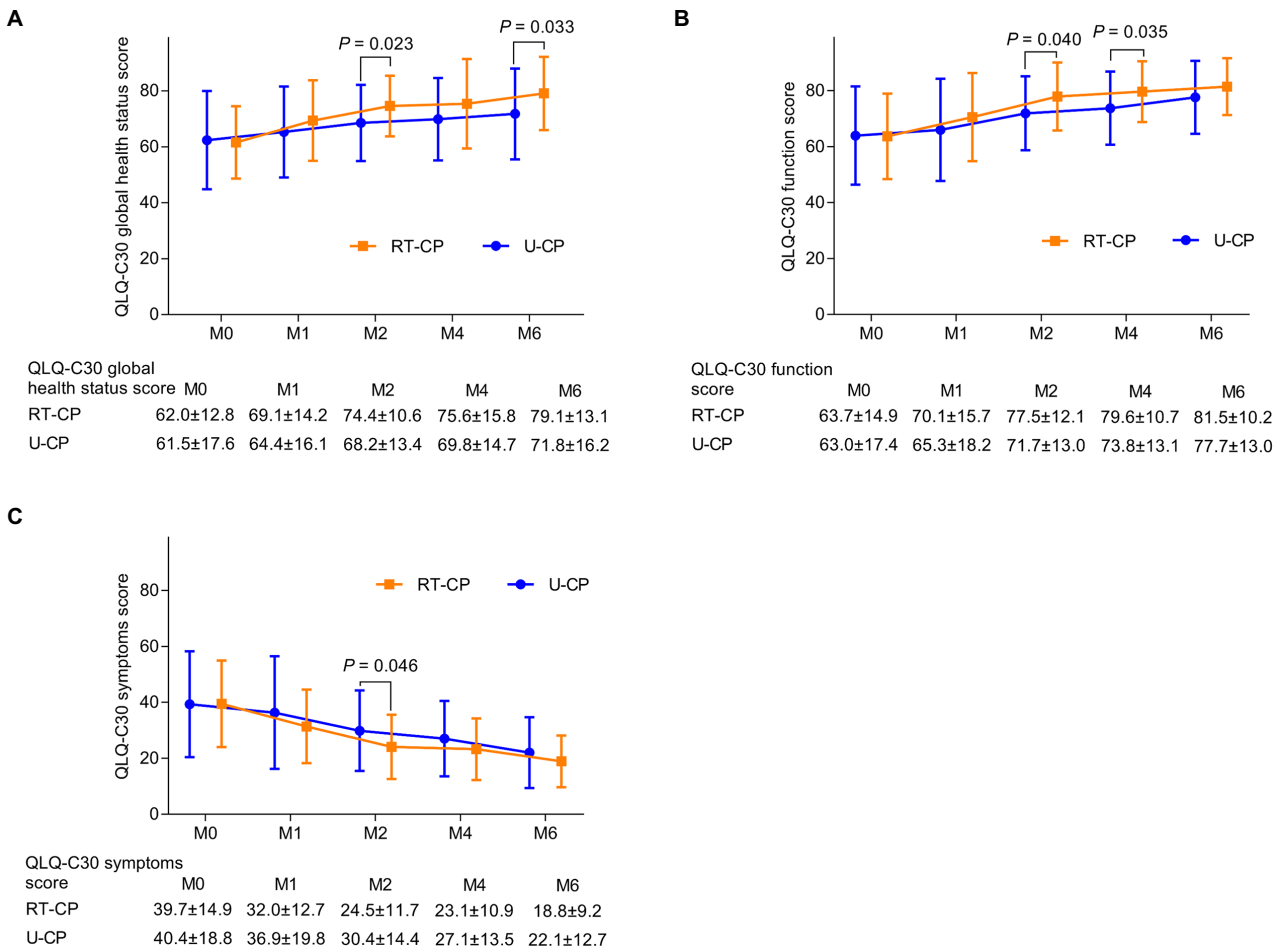
Comparison of QLQ-C30 score at different time points between RT-CP group and U-CP group. QLQ-C30 global health score at M2 and M6 was increased in the RT-CP group compared with the U-CP group **(A)**; QLQ-C30 function score at M2 and M4 was elevated in the RT-CP group compared to the U-CP group **(B)**; QLQ-C30 symptom score at M2 was reduced in the RT-CP group in contrast with the U-CP group **(C)**.

Quality of Life Questionnaire-Core 30 global health status score was enhanced at M2 (74.4 ± 10.6 vs. 68.2 ± 13.4; *p* = 0.023) and M6 (79.1 ± 13.1 vs. 71.8 ± 16.2; *p* = 0.033) in the RT-CP group by comparison with the U-CP group; while it was not different at M0 (62.0 ± 12.8 vs. 61.5 ± 17.6), M1 (69.1 ± 14.2 vs. 64.4 ± 16.1), and M4 (75.6 ± 15.8 vs. 69.8 ± 14.7) between the RT-CP group and U-CP group (all *p* > 0.05; Figure 3A). Meanwhile, the QLQ-C30 function score was increased at M2 (77.5 ± 12.1 vs. 71.7 ± 13.0; *p* = 0.040) and M4 (79.6 ± 10.7 vs. 73.8 ± 13.1; *p* = 0.035) in the RT-CP group compared to the U-CP group; whereas it did not differ at M0 (63.7 ± 14.9 vs. 63.0 ± 17.4), M1 (70.1 ± 15.7 vs. 65.3 ± 18.2), and M6 (81.5 ± 10.2 vs. 77.7 ± 13.0) between the RT-CP group and U-CP group (all *p* > 0.05; Figure 3B). Furthermore, the QLQ-C30 symptoms score was reduced at M2 (24.5 ± 11.7 vs. 30.4 ± 14.4; *p* = 0.046) in the RT-CP group in contrast with the U-CP group but did not differ at M0 (39.7 ± 14.9 vs. 40.4 ± 18.8), M1 (32.0 ± 12.7 vs. 36.9 ± 19.8), M4 (23.1 ± 10.9 vs. 27.1 ± 13.5), and M6 (18.8 ± 9.2 vs. 22.1 ± 12.7) between the RT-CP group and U-CP group (all *p* > 0.05; Figure 3C).

### Comparison of survival between RT-CP group and U-CP group

**Figure 4:**
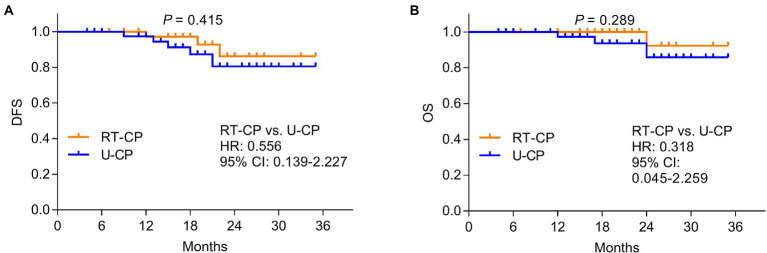
Comparison of DFS and OS between RT-CP group and U-CP group. disease-free survival (DFS) **(A)** and overall survival (OS) **(B)** were not different between the RT-CP group and the U-CP group.

Further comparison analysis was conducted to evaluate the potential survival benefit of RT-CP in older PTC patients. However, it was discovered that DFS was not different between the RT-CP group and U-CP group (*p* = 0.415; Figure 4A); meanwhile, OS also did not differ between the RT-CP group and U-CP group (*p* = 0.289; Figure 4B).

### Subgroup analysis of outcomes at M6 between RT-CP group and U-CP group

In female older PTC patients, the HADS-A score (*p* = 0.016) and HADS-D score (*p* = 0.024) were decreased, while the QLQ-C30 global health status score (*p* = 0.049) was increased in the RT-CP group compared to the U-CP group. In single/divorced/widowed older PTC patients, the HADS-A score (*p* = 0.003) and HADS-D score (*p* = 0.004) were decreased, while the QLQ-C30 global health status score (*p* = 0.010) was increased in the RT-CP group compared to the U-CP group. In older PTC patients with the pTNM stage of I/II, the HADS-A score was decreased in the RT-CP group compared to the U-CP group (*p* < 0.001; Table 4).

**Table 4:** Subgroup analysis of outcomes at M6 among the older PTC patients.

Items	RT-CP	U-CP	*p*-value
Gender			
Male			
HADS-A score^a^	5.5 ± 2.2	5.9 ± 2.8	0.700
HADS-D score^a^	5.5 ± 2.3	6.6 ± 2.7	0.297
QLQ-C30 global health status score^a^	80.2 ± 11.9	74.9 ± 14.2	0.336
QLQ-C30 functions score^a^	81.3 ± 9.5	77.8 ± 14.2	0.492
QLQ-C30 symptoms score^a^	19.6 ± 8.0	17.9 ± 10.0	0.637
Female			
HADS-A score^a^	5.3 ± 2.8	7.1 ± 2.5	0.016
HADS-D score^a^	5.1 ± 2.1	6.5 ± 2.4	0.024
QLQ-C30 global health status score^a^	78.6 ± 13.8	70.0 ± 17.3	0.049
QLQ-C30 functions score^a^	81.6 ± 10.6	77.6 ± 12.6	0.214
QLQ-C30 symptoms score^a^	18.5 ± 9.7	24.4 ± 13.5	0.069
Marital status			
Single/Divorced/Widowed			
HADS-A score^a^	5.0 ± 2.4	6.9 ± 2.8	0.003
HADS-D score^a^	5.0 ± 2.1	6.8 ± 2.7	0.004
QLQ-C30 global health status score^a^	80.5 ± 12.7	70.8 ± 16.4	0.010
QLQ-C30 functions score^a^	81.4 ± 10.2	76.6 ± 13.6	0.116
QLQ-C30 symptoms score^a^	18.0 ± 9.3	21.8 ± 12.4	0.168
Married			
HADS-A score^a^	7.0 ± 3.3	5.4 ± 1.6	0.276
HADS-D score^a^	6.0 ± 2.4	5.4 ± 1.0	0.568
QLQ-C30 global health status score^a^	72.4 ± 14.1	76.1 ± 16.1	0.654
QLQ-C30 functions score^a^	82.1 ± 10.9	82.6 ± 9.3	0.938
QLQ-C30 symptoms score^a^	22.6 ± 8.2	23.4 ± 14.8	0.896
pTNM stage			
I/II			
HADS-A score^a^	4.8 ± 2.4	7.6 ± 2.4	<0.001
HADS-D score^a^	5.0 ± 2.1	6.3 ± 2.5	0.063
QLQ-C30 global health status score^a^	79.0 ± 12.0	71.0 ± 17.5	0.073
QLQ-C30 functions score^a^	80.7 ± 9.6	78.4 ± 12.2	0.476
QLQ-C30 symptoms score^a^	18.8 ± 8.4	23.6 ± 14.3	0.170
III/IV			
HADS-A score^a^	6.1 ± 2.7	4.9 ± 2.2	0.204
HADS-D score^a^	5.4 ± 2.3	7.0 ± 2.5	0.087
QLQ-C30 global health status score^a^	79.2 ± 15.0	73.2 ± 14.3	0.275
QLQ-C30 functions score^a^	82.7 ± 11.1	76.4 ± 14.7	0.192
QLQ-C30 symptoms score^a^	18.8 ± 10.4	19.4 ± 8.9	0.867

PTC, papillary thyroid carcinoma; M6, six months discharge after operation; RT-CP, reminiscence therapy care program; U-CP, usual care program; HADS-A, Hospital Anxiety and Depression Scale for anxiety; HADS-D, Hospital Anxiety and Depression Scale for depression; QLQ-C30, Quality of Life Questionnaire-Core 30; pTNM, pathological-tumor-node-metastasis. ^a^Variables described by mean ± SD.

## Discussion

In recent years, RT has achieved a lot of attention for mitigating anxiety and depression in cancer patients (Zhao, 2021; Huang et al., 2022). A study claims that RT is effective for glioma patients to reduce anxiety and depression (Zhao, 2021). Besides, RT could serve as a potential nursing intervention for attenuating anxiety and depression in prostate cancer patients (Huang et al., 2022). However, the potency of RT in older PTC patients requires to be explored. This study discovered that anxiety and depression were attenuated to some extent in the RT-CP group compared with the U-CP group. The potential reasons might be that: (Megwalu and Moon, 2022) RT-CP encouraged older PTC patients to recall and share past positive memories, which would make them feel valued and connected (Liu and Li, 2021; Zhao, 2021; Sanabria et al., 2022) older PTC patients were likely to develop negative emotions, such as lonely, abandoned, unhappy, self-isolated, etc., while RT-CP allowed them to share pleasant memories with others, which helped these patients to regain hope, confidence, and self-esteem, thus helping them to have an optimistic mindset towards life (Li et al., 2022). Thereby RT-CP could eliminate anxiety and depression to some extent in older PTC patients. Moreover, anxiety rate and depression rate were only slightly decreased in the RT-CP group by comparison with the U-CP group but did not reach statistical significance. The potential argument would be that: the sample size was relatively insufficient in this study, which led to low statistical power.

In addition to anxiety and depression, RT also plays an essential role in improving the quality of life in cancer patients, including patients with NSCLC, gastric cancer, glioma, colorectal cancer, etc. (Liu and Li, 2021; Zhang et al., 2021; Zhao, 2021; Zhou and Sun, 2021). This study observed that: (Megwalu and Moon, 2022) RT-CP could improve the global health status to a certain extent in older PTC patients. The possible reason might be that: as mentioned above, RT-CP allowed older PTC patients to share wonderful memories with other patients, which would help patients to rebuild self-identity, enhance social ability, make new friends, and trigger the aspiration for a better life (Liu and Li, 2021; Zhang et al., 2021; Zhao, 2021); all of these helped them to build a positive attitude towards life, and might further improve the quality of life. (Sanabria et al., 2022) RT-CP could also enhance the function score to some extent. The possible explanation would be that: as discussed above, RT-CP could improve patients’ mental health by recalling and sharing pleasant memories with others, which helped them to restore emotional, cognitive, and social functions; additionally, RT-PC also provided health education, which might assist patients in restoring physical and role functions (Liu and Li, 2021; Zhao, 2021; Zhou and Sun, 2021; Li et al., 2022). Taking these together, the function score was also increased in the RT-CP group compared to the U-CP group. (McDow et al., 2021) The symptom score was decreased in the RT-CP group in contrast with the U-CP group. The possible argument would be that: RT-CP helped patients to interact with others, which might allow them to better understand how to deal with symptoms, including fatigue, pain, nausea, and vomiting (Zhao, 2021; Huang et al., 2022; Li et al., 2022). However, health education was also provided in the U-CP group. Hence, RT-CP could only reduce symptom scores to some extent in older PTC patients compared to U-CP.

Meanwhile, this study also explored the effect of RT-CP on survival in older PTC patients. It was found that DFS and OS were not different between the RT-CP group and the U-CP group. The possible argument would be that: the determinants of prognosis mainly included age, tumor size, metastasis, thyroid gland invasion, etc.; therefore, the improvement of mental health and quality of life might have a minor or even no effect on the survival profile in older PTC patients (Shin et al., 2020; Sun et al., 2020).

Further subgroup analysis suggested that the effect of RT-CP was more obvious in female, single/divorced/widowed, and pTNM stage of I/II older PTC patients. The potential reasons would be that: (Megwalu and Moon, 2022) female and single/divorced/widowed patients were more likely to suffer from emotional or psychological problems, such as anxiety and depression (Hald et al., 2022); as discussed above, RT-CP could effectively ameliorate anxiety and depression; thus, anxiety and depression were decreased in female, single/divorced/widowed patients receiving RT-CP compared to those receiving U-CP; (Sanabria et al., 2022) the disease conditions were complicated and severe in patients with higher pTNM stage, indicating that the dysregulation of thyroid hormones was more aggravated; therefore, brain function might be gravely affected, which would reduce the effect of RT-CP in these patients (Gobel et al., 2020). As a result, RT-CP could only attenuate HADS-A score in older PTC patients with pTNM stage of I/II.

Notably, considering that premorbid anxiety and depression would affect postoperative mental issues, it was necessary to make a clear statement to clarify this issue. (Megwalu and Moon, 2022) This problem had been noticed before the current research started; therefore, the corresponding exclusions were set for patients who had mental diseases (including anxiety and depression), and for patients who had documented diagnoses of anxiety and depression before surgery. (Sanabria et al., 2022) To avoid further misunderstanding, it also should be noticed that this study only excluded patients who had documented diagnoses of anxiety and depression, while did not exclude patients who had symptoms of anxiety and depression before the surgery. Since the base of older PTC patients is relatively fewer compared with young adults, and if the patients who had preoperative symptoms of anxiety and depression were strictly excluded, then the number of eligible subjects would be very small.

Several limitations should be noticed in this study: (Megwalu and Moon, 2022) the follow-up duration was not long enough; thus, the long-term effect of RT-CP on improving anxiety, depression, and quality of life in older PTC patients should be further explored; (Sanabria et al., 2022) this was a single-center study; therefore, selection bias might exist; (McDow et al., 2021) HADS score was self-assessed; therefore, assessment bias would exist in this study.

In summary, RT-CP may be a potential intervention for ameliorating anxiety and depression, as well as improving the quality of life, while it has no effect on prolonging survival in older PTC patients. Clinically, RT-CP as a non-pharmacological intervention can be effective in improving psychiatric problems and quality of life in older PTC patients; however, the intervention approach taken in this study is conducted in a group setting; thus, whether the individual model of this intervention is equally effective or more effective for older PTC patients requires further research to verify.

## Data availability statement

The original contributions presented in the study are included in the article/supplementary material, further inquiries can be directed to the corresponding author.

## Ethics statement

The studies involving human participants were reviewed and approved by Ethics Committee of Harbin Medical University Cancer Hospital. The patients/participants provided their written informed consent to participate in this study.

## Author contributions

YL conceived and designed the experiment, analyzed the data, and revised the manuscript. LC collected and analyzed the data. XY performed data analysis and provided interpretation. XR provided technical support and analyzed and interpreted the results. All authors contributed to the article and approved the submitted version.

## Conflict of interest

The authors declare that the research was conducted in the absence of any commercial or financial relationships that could be construed as a potential conflict of interest.

## Publisher’s note

All claims expressed in this article are solely those of the authors and do not necessarily represent those of their affiliated organizations, or those of the publisher, the editors and the reviewers. Any product that may be evaluated in this article, or claim that may be made by its manufacturer, is not guaranteed or endorsed by the publisher.
